# Microbial Interactions Drive Distinct Taxonomic and Potential Metabolic Responses to Habitats in Karst Cave Ecosystem

**DOI:** 10.1128/Spectrum.01152-21

**Published:** 2021-09-08

**Authors:** Liyuan Ma, Xinping Huang, Hongmei Wang, Yuan Yun, Xiaoyu Cheng, Deng Liu, Xiaolu Lu, Xuan Qiu

**Affiliations:** a School of Environmental Studies, China University of Geosciences, Wuhan, China; b State Key Laboratory of Biogeology and Environmental Geology, China University of Geosciences, Wuhan, China; c College of Life Sciences, Nankai University, Tianjin, China; University of Minnesota

**Keywords:** cooccurrence network, functional prediction, karst cave, keystone species, microbial interactions

## Abstract

The geological role of microorganisms has been widely studied in the karst cave ecosystem. However, microbial interactions and ecological functions in such a dark, humid, and oligotrophic habitat have received far less attention, which is crucial to understanding cave biogeochemistry. Herein, microorganisms from weathered rock and sediment along the Heshang Cave depth were analyzed by random matrix theory-based network and Tax4Fun functional prediction. The results showed that although the cave microbial communities have spatial heterogeneity, differential habitats drove the community structure and diversity. *Actinobacteria* were predominant in weathered rock, whereas *Proteobacteria* dominated the sediment. The sediment communities presented significantly higher alpha diversities due to the relatively abundant nutrition from the outside by the intermittent stream. Consistently, microbial interactions in sediment were more complex, as visualized by more nodes and links. The abundant taxa presented more positive correlations with other community members in both of the two networks, indicating that they relied on promotion effects to adapt to the extreme environment. The keystones in weathered rock were mainly involved in the biodegradation of organic compounds, whereas the keystone *Nitrospira* in sediment contributed to carbon/nitrogen fixation. Collectively, these findings suggest that microbial interactions may lead to distinct taxonomic and functional communities in weathered rock and sediment in the subsurface Heshang Cave.

**IMPORTANCE** In general, the constant physicochemical conditions and limited nutrient sources over long periods in the subsurface support a stable ecosystem in karst cave. Previous studies on cave microbial ecology were mostly focused on community composition, diversity, and the relationship with local environmental factors. There are still many unknowns about the microbial interactions and functions in such a dark environment with little human interference. Two representative habitats, including weathered rock and sediment in Heshang Cave, were selected to give an integrated insight into microbial interactions and potential functions. The cooccurrence network, especially the subnetwork, was used to characterize the cave microbial interactions in detail. We demonstrated that abundant taxa primarily relied on promotion effects rather than inhibition effects to survive in Heshang Cave. Keystone species may play important metabolic roles in sustaining ecological functions. Our study provides improved understanding of microbial interaction patterns and community ecological functions in the karst cave ecosystem.

## INTRODUCTION

Karst area is a strategic zone for research related to climate change and carbon flux because the unique terrestrial cave is subject to changes in response to external environmental conditions (1, 2). The karst cave is characterized by darkness and dampness with “buffering effects,” where microorganisms play critical roles in regulating the regional biogeochemical cycles (3, 4). It has been proposed that cave microorganisms were translocated soil heterotrophs, which were transferred into the cave by air currents or water percolating from the terrestrial surface (5). These exotic microorganisms were subsequently adapted to different habitats in caves, such as weathered rock, sediment, stalagmite, and drip water (6, 7).

In recent years, with the rapid development of high-throughput sequencing technology, the dominant microbial species and their functions in caves have been gradually discovered (8, 9). The cave microbial communities exhibited high taxonomic diversity (10, 11). It has been reported that the microbial communities in Kartchner caverns (eastern North America) and Jinjia caves (eastern Asia) were similar, despite the differential geographical locations (12, 13). In particular, caves harbored a wide variety of mineral-utilizing microorganisms that figured prominently in the formation of secondary mineral deposits and unusual mineralized microstructures recognized as biosignatures (14, 15). Genomic evidence showed that microbial communities specifically adapted to the low-nutrient and high-calcium conditions in carbonate caves (16), while the survival of a microbial slime community was driven by inorganic nitrogen metabolism (6). In the subterranean cave environment, microbial living activities and their interactions were critical in maintaining community stability and ecosystem functioning (17, 18). The isolated cave ecosystem has been proposed as a model system for advancing microbial ecology (19). However, our understanding about the microbial interactions in the shallow subsurface of karst caves is still very limited.

Molecular ecological network (MEN) models provide powerful ways to approximate the interactions of multiple microorganisms in different ecosystems (20, 21), especially for the uncultured rare species in natural habitats. The random matrix theory (RMT)-based network construction method has been proven due to the advantages of strong reliability, high sensitivity, and robustness for complex communities (22). Recently, microbial ecologists have successfully explored the symbiotic pattern of microbial communities by using network analysis based on their correlations in natural habitats, such as large spatial scale soils (20), interconnected rhizosphere (23), and contaminated area (22). The network graphs, which were used to describe the cooccurrence patterns between operational taxonomic units (OTUs) across different samples but not necessarily their spatial contact and interactions, were built to represent positive and negative relationships (24). Positive interactions usually referred to promotion effects, including commensalism and mutualism, while negative interactions referred to inhibition effects, including parasitism, antagonism, and predation (25, 26). Additionally, putative keystone taxa can be identified from the network for subsequent analysis of community stability and functions (27, 28). Typically, microbial trophic structures in caves were considered not as complex as their counterparts in general terrestrial environments due to the absence of light, the limited nutrition, and the reduced fluctuations in temperature and humidity (29). Previous studies on show caves have demonstrated that most bacterial interactions were positive based on cooccurrence network analysis (18, 30), but how about the microbial interactions with little human disturbance? How do microbial interactions affect the potential community functions in such a model system? Understanding how these extraordinary assemblages respond and adapt to the harsh conditions is urgently needed (31, 32).

The undeveloped Heshang Cave is located in western Hubei Province. Environmental conditions within the cave have been continuously monitored since 2004 (33–35). Our previous work has confirmed that pH and total organic carbon (TOC) were the deterministic factors affecting the distribution of microbial communities in Heshang Cave (36). Herein, this study focused on the weathered rock (W) and sediment (S), where microbial long-term succession and interactions resulted in particularly stable communities. The vertical distributions of dominant genera were assessed. Additionally, molecular ecological networks of microbial communities from the two habitats were constructed to reveal the interactions of cave microbes. Based on the above analysis, an integrated insight into the microbial interactions in two habitats of the cave was proposed by addressing the following descriptors: (i) taxonomic composition of communities and spatial distribution characteristics, (ii) microbial interaction patterns in weathered rock and sediment, and (iii) microbial contribution to the cave ecosystem functioning.

## RESULTS

### Microbial community distribution patterns.

**FIG 1:**
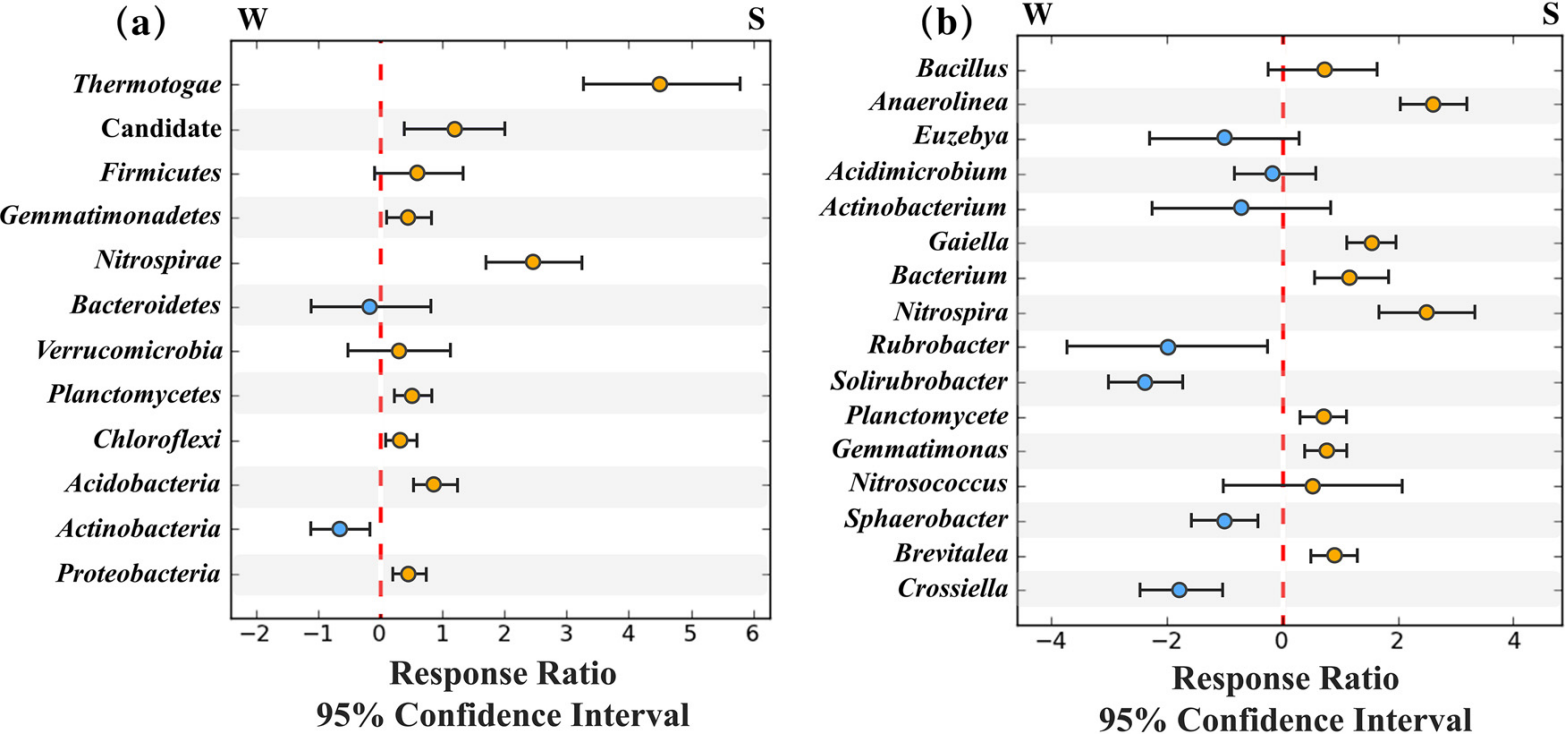
Response ratio of microbial relative abundances in sediment to weathered rock at the phylum level (a) and at the genus level (b) with 95% confidence. The blue points represent weathered rock, and the orange points represent sediment.

The pH of weathered rock was significantly lower than that of sediment, while the TOC, Ca^2+^, K^+^, and NH_4_^+^ in weathered rock were all considerably higher (*P* < 0.05) than those in sediment (Table 1), suggesting a habitat specificity between the weathered rock and sediment. Microorganisms in the two habitats generally differentiated and developed their own characteristics. The sediment community showed a significantly higher diversity than weathered rock (*P* < 0.05) in terms of Shannon-Wiener index (*H*), Pielou evenness (*J*), and OTU richness (Table 2). A response ratio analysis of the top 12 phyla (97.80%) was conducted, showing that *Actinobacteria* was more abundant in weathered rock, whereas *Proteobacteria*, *Acidobacteria*, *Chloroflexi*, *Planctomycetes*, *Nitrospirae*, Candidate (candidate phylum radiation [CPR]), and *Thermotogae* were more abundant in sediment (Fig. 1a). As to the top 16 genera (50.30%), the relative abundances of *Crossiella*, *Sphaerobacter*, *Solirubrobacter*, and *Rubrobacter* were higher in weathered rock, while the relative abundances of *Brevitalea*, *Gemmatimonas*, *Planctomycete*, *Nitrospira*, *Bacterium*, *Gaiella*, and *Anaerolinea* were higher in sediment (Fig. 1b). Based on Pearson’s correlation coefficient (*r*) calculation, *Acidobacteria*, *Planctomycetes*, and *Verrucomicrobia* showed significant correlations with pH and TOC simultaneously (Table S1 in the supplemental material). All the evidence indicated that the cave microorganisms in weathered rock and sediment had obvious habitat specificity.

**TABLE 1:** Physicochemical parameters from weathered rock (W) and sediment (S) of Heshang Cave

	Weathered rock (W)^*a*^	Sediment (S)^*a*^
pH	7.79 ± 0.22 b	**8.09 ± 0.20 a**
TOC (%)	**1.00 ± 0.82 a**	0.39 ± 0.20 b
Ca^2+^ (mM)	**4.30 ± 5.68 a**	1.99 ± 1.93 b
Mg^2+^ (mM)	8.97 ± 18.24 a	3.74 ± 4.44 a
K^+^ (mM)	**0.99 ± 0.98 a**	0.20 ± 0.13 b
Na^+^ (mM)	0.50 ± 0.62 a	0.51 ± 0.63 a
NH_4_^+^ (mM)	**0.44 ± 0.48 a**	0.06 ± 0.10 b
Cl^−^ (mM)	1.41 ± 1.30 a	1.40 ± 2.02 a
NO_2_^−^ (mM)	2.25 ± 3.79 a	0.37 ± 0.74 a
NO_3_^−^ (mM)	6.44 ± 9.14 a	3.62 ± 4.80 a
SO_4_^2−^ (mM)	10.47 ± 17.65 a	0.98 ± 1.50 a

a Values are mean ± standard error. Different small letters within the same row indicate a significant difference at the level of 0.05. The bold formatting indicates a significant difference between W and S.

**TABLE 2:** Alpha diversity indexes of the microbial communities in weathered rock (W) and sediment (S)

Community	Shannon-Wiener index (*H*)^*a*^	Pielou evenness index (*J*)^*a*^	OTU richness^*a*^
Weathered rock (W)	4.91 ± 0.41 b	0.63 ± 0.04 b	2,333.9 ± 397.77 b
Sediment (S)	**6.21 ± 0.62 a**	**0.77 ± 0.06 a**	**3,329.3 ± 479.01 a**

a Values are means ± standard error. Different small letters indicate significant differences at the level of 0.05. The bold formatting indicates a significant difference between W and S.

**FIG 2:**
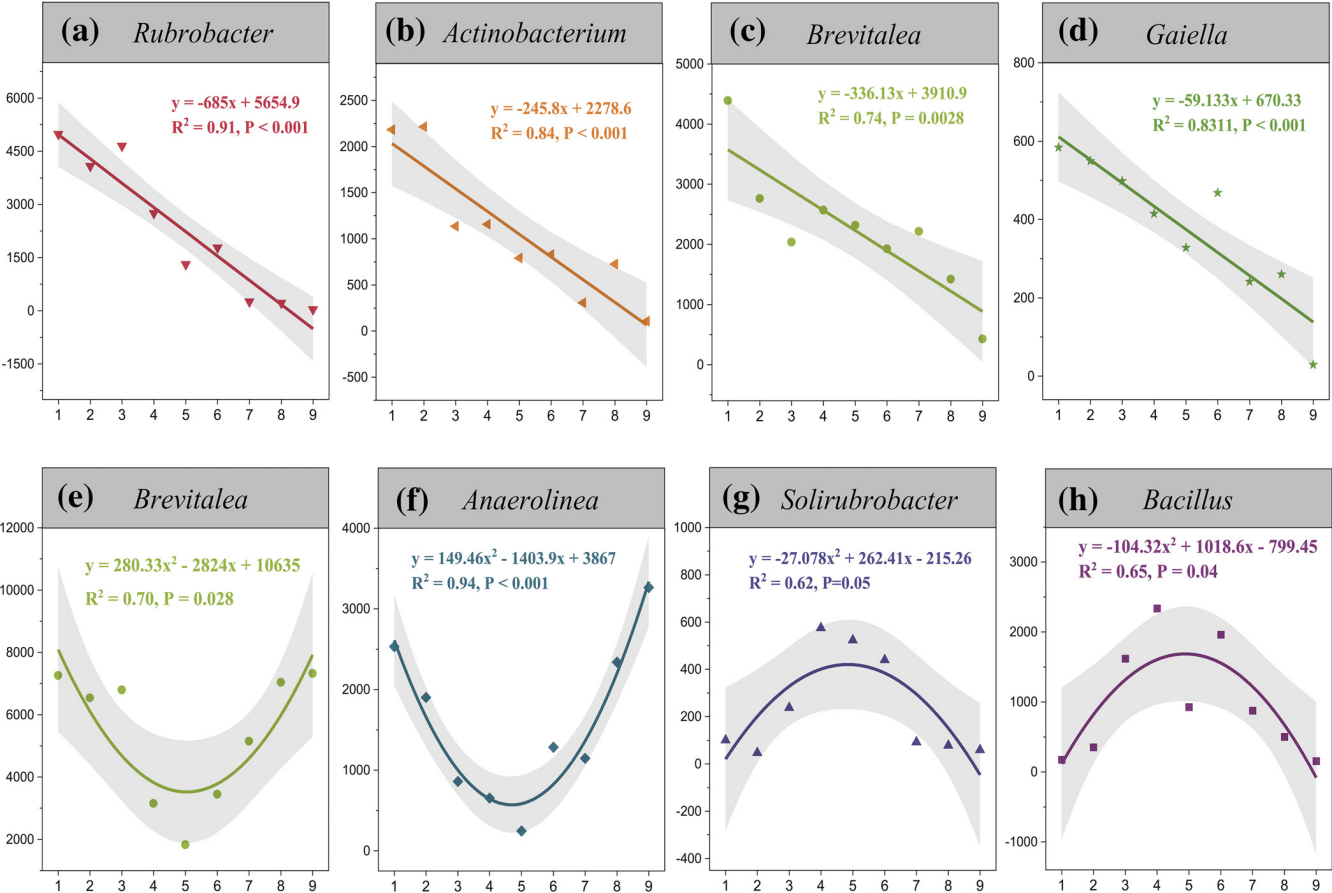
Linear regression analyses of dominant genera in weathered rock, including *Rubrobacter* (a), *Actinobacterium* (b), *Brevitalea* (c), and *Gaiella* (d), and nonlinear regression analyses of dominant genera in sediment, including *Brevitalea* (e), *Anaerolinea* (f), *Solirubrobacter* (g), and *Bacillus* (h). The numbers from 1 to 9 represent the sampling site from the cave entrance (1) to the deep-cave zone (9). The y axis represents tags (#).

The dominant genera were also different from the entrance to the deep-cave zone. The dominant genera, such as *Rubrobacter*, *Actinobacterium*, *Brevitalea*, and *Gaiella*, showed strong linear relationships with the cave depth in weathered rock (*R*^2^ > 0.74, *P* < 0.01) (Fig. 2). For example, *Rubrobacter* decreased from the entrance to the deep cave with an *R*^2^ up to 0.91. By contrast, the variations of dominant genera in sediment were bell shaped (*Solirubrobacter* and *Bacillus*) or like an inverted bell jar (*Brevitalea* and *Anaerolinea*) (Fig. 2), which were affected by the large stone flower pool that changed the flow directions of the intermittent stream (Fig. S1). There were 5,167 core OTUs identified in weathered rock and sediment; nevertheless, only 2,288 and 3,119 core OTUs were shared from the entrance to the deep zone in their respective habitats (Fig. S2). The number of unique OTUs at the entrance or at the deep zone also proved the spatial heterogeneity of microbial distribution in Heshang Cave. Taken together, cave microorganisms in weathered rock and sediment showed notable correlations to their geological locations.

**FIG 3:**
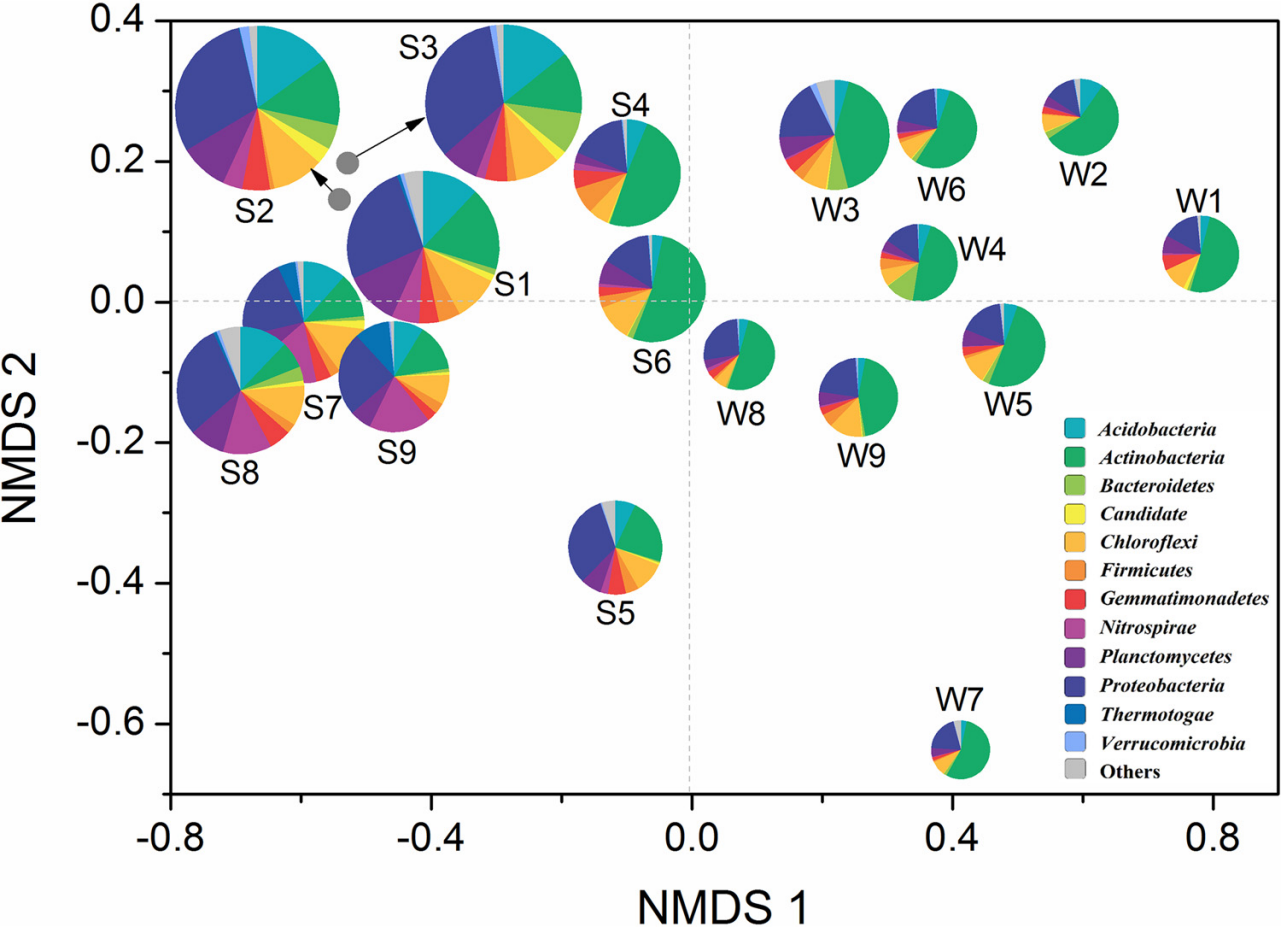
Comprehensive insight into the divergence between microbial communities in weathered rock and sediment in Heshang Cave; radius, alpha diversity (*H*); distribution, beta diversity; proportion, relative abundance at the phylum level.

To give a comprehensive insight into the divergence of microbial communities from the two habitats, a nonmetric multidimensional scaling (NMDS) analysis was performed to examine the biological similarity patterns with respect to benthic assemblages among sites (Fig. 3). Shannon index acted as the radius of the pie, and the microbial communities were presented at the phylum level. As expected, the Shannon indexes of S4, S5, and S6 were lower than those in other sediment communities, and the abundances of *Actinobacteria* in S4, S5, and S6 were much higher, which were a little more similar to the weathered rock samples. As a whole, the sediment samples were clustered together at the negative half of the *x* axis, whereas the weathered rock samples converged at the positive half. The microbial communities maintained similar composition, diversity, and dominant species under the same habitat condition. These findings indicated that the microbial community composition in weathered rock and sediment differed from each other in Heshang Cave. Therefore, the cave habitat, rather than the cave depth, served as the basis for our subsequent network grouping.

### Cooccurrence network characteristics.

To understand the cooccurrence of microbial populations in Heshang Cave, RMT-based molecular ecological networks for weathered rock and sediment were constructed. In total, 5,575 and 6,452 OTUs were retained in W and S networks, respectively (Table 3). With the similarity thresholds of 0.930 (W) and 0.960 (S), more nodes and links were observed in the S network (1,269 nodes, 2,097 links) than in the W network (1,149 nodes, 1,488 links). Additionally, there were 1,513 (72.15%) positive interactions shown in blue lines in the S network, while only 925 (62.16%) positive interactions were in the W network. The topology of the two networks fit very well with the power law distribution. As a result, the *R*^2^ of power law achieved 0.936 (W) and 0.917 (S), indicating that the two networks displayed scale-free features. These network characteristics suggested that the network structures were nonrandom and unlikely to change substantially.

**TABLE 3:** The major topological properties of the empirical networks of microbial communities in weathered rock and sediment

Community	No. of OTUs	Total nodes	Total links	St^*a*^	*R*^2^ of power law	AvgK^*a*^	GD^*a*^	AvgCC^*a*^	Module	Modularity
Weathered rock (W)	5,575	1,149	1,488	0.930	0.936	2.590	8.794	0.077	148	0.893
Sediment (S)	6,452	1,269	2,097	0.960	0.917	3.305	6.688	0.099	215	0.740

a St, similarity threshold; AvgK, average connectivity; GD, average path distance; AvgCC, average clustering coefficient.

**FIG 4:**
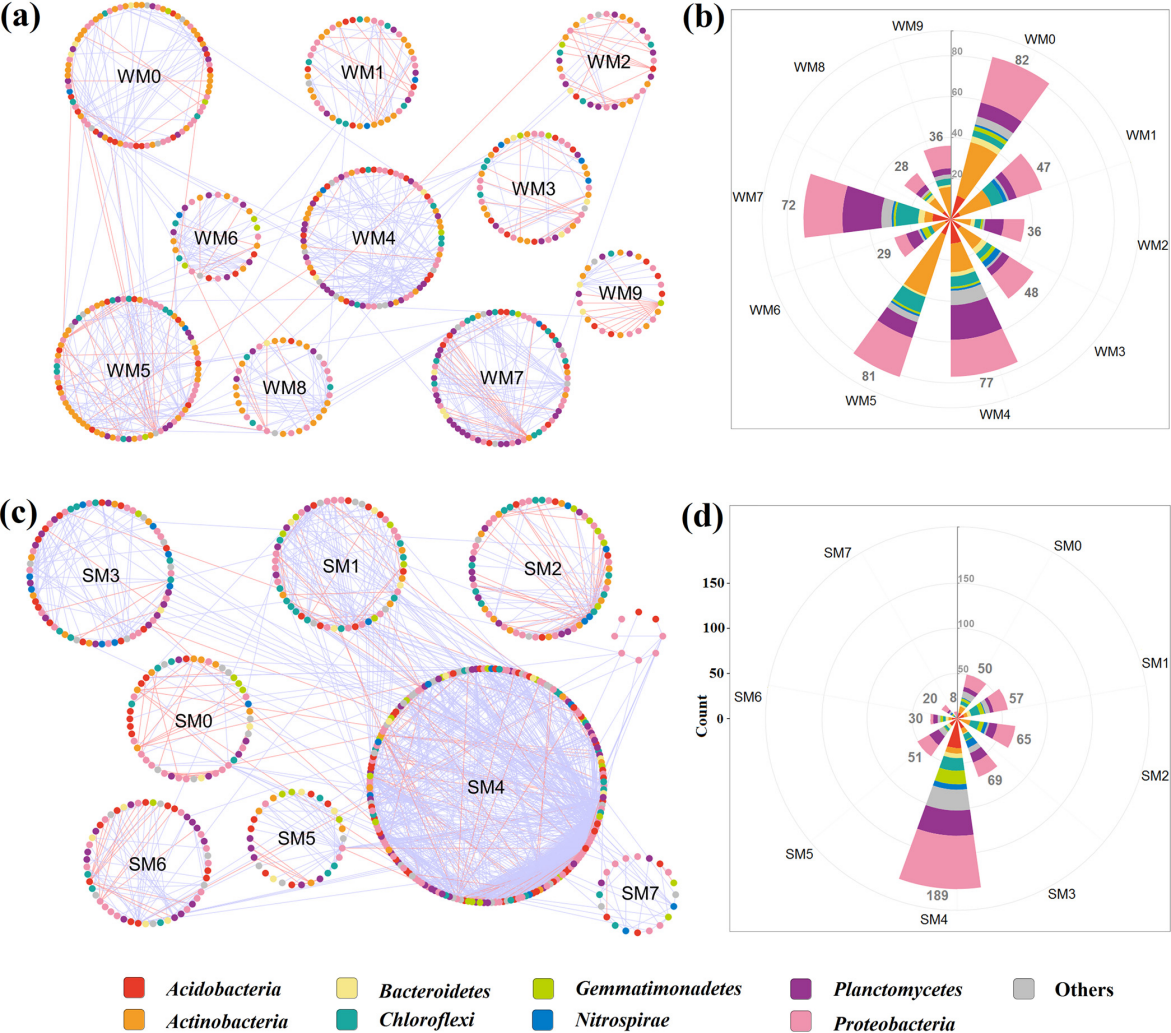
Highly connected modular organization of the pMENs of weathered rock (a, b) and sediment (c, d). Node color indicates different major phyla. The blue lines indicate positive interactions, and red lines indicate negative interactions. Rose diagrams (b and d) represent the composition of modules with greater than 8 nodes.

The average path distance of the W network was 8.794, while the average path distance of the S network was 6.688, suggesting that the OTUs in the S network were more closely connected than those in the W network. The modularity of the W network was 0.893, which was higher than 0.740 of the S network. In total, 148 modules in W and 215 modules in S were generated, within which the nodes numbered higher than 8 were regarded as major modules. As a result, 536 and 539 nodes were visualized in the weathered rock and sediment networks, respectively (Fig. 4a and c). The module sizes of the W network were relatively homogeneous, ranging from 28 to 82 in each rose leaf. However, the module sizes of the S network were smaller than 69, except a large one (SM4) achieved 189 (Fig. 4b and d). The different composition of modules in networks was also apparent from the rose diagrams. *Actinobacteria* dominated the two largest modules of WM0 and WM5 and primarily cooccurred with *Proteobacteria*. *Proteobacteria* seemed to be prominent members of all the W modules, and cooccurred with *Planctomycetes* in WM4 and WM7. By contrast, *Proteobacteria* also acted as the dominant phylum in SM4, where it cooccurred with *Planctomycetes*, *Acidobacteria*, and *Gemmatimonadetes*.

**FIG 5:**
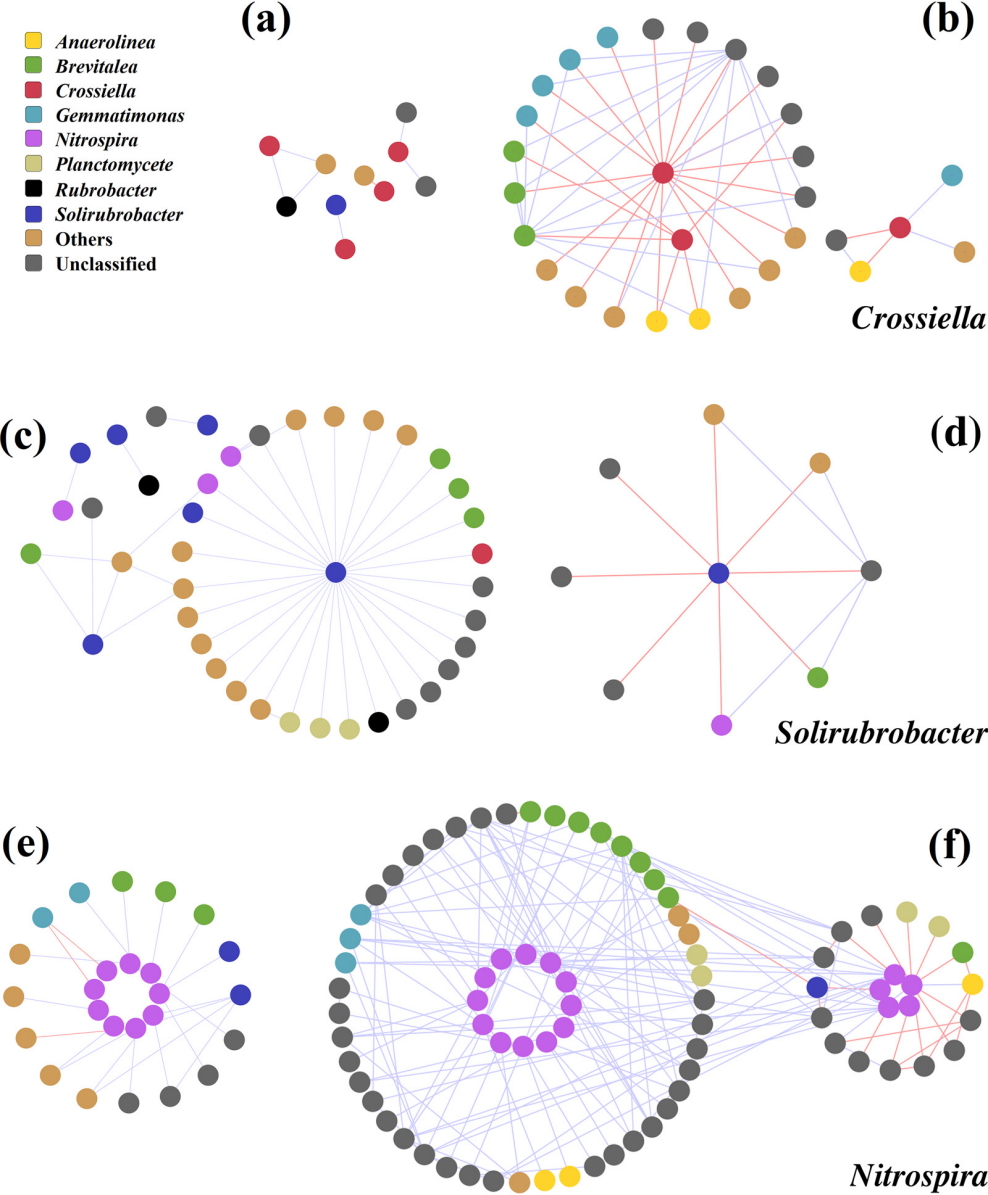
Network interactions of the abundant genera in weathered rock and sediment. (a, b) Representative genus *Crossiella* in the W and S networks. (c, d) *Solirubrobacter* in W and S networks. (e, f) *Nitrospira* in W and S networks. Node color indicates different genera. The blue lines indicate positive interactions, and red lines indicate negative interactions.

Subnetworks for the representative dominant genera were further constructed to visualize their possible interactions in communities (Fig. 5). *Crossiella* and *Solirubrobacter* were significantly abundant in the weathered rock (Fig. 1b). As expected, *Crossiella* was preserved in the W network by more positive links, while it presented almost all negative interactions with *Gemmatimonadetes*, *Brevitalea*, *Anaerolinea*, and other genera in the S network (Fig. 5a and b). All the detected links of *Solirubrobacter* showed positive interactions in weathered rock; however, all of those in sediment were negative (Fig. 5c and d). In contrast, *Nitrospira* was more competitive, with more complex interactions in sediment than in weathered rock, as evidenced by more nodes and links (Fig. 5e and f). This evidence indicated that the abundant taxa were positively correlated with other populations in their respective habitats.

### Putative keystone taxa.

**FIG 6:**
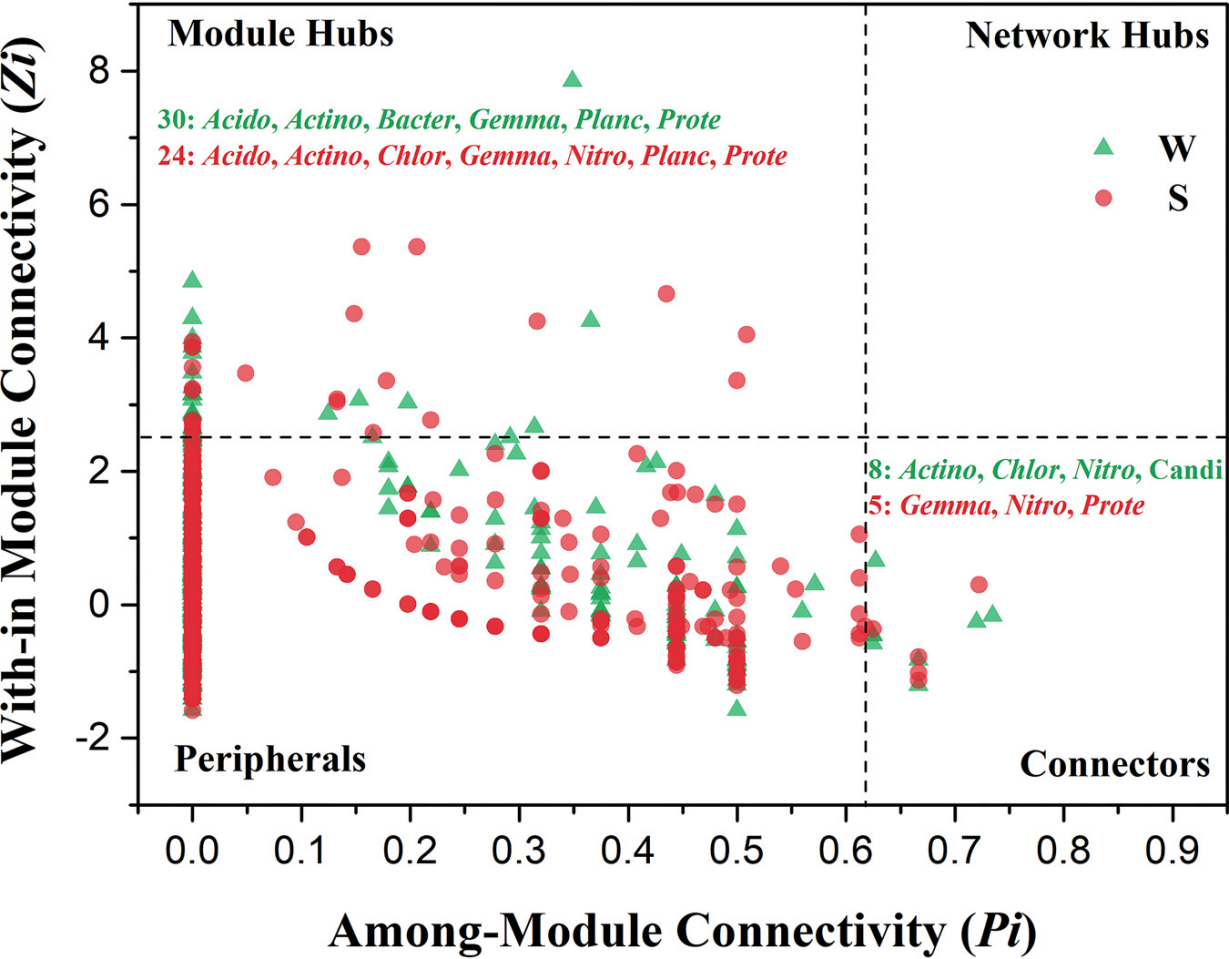
Classification of nodes to identify putative keystone species within the networks of weathered rock (W) and sediment (S). Each symbol represents an OTU from the two-network detailed module analyses (Fig. 4). Module hubs have a *Zi *of >2.5, whereas connectors have a *Pi *of >0.62. The number of module hubs and connectors is shown on the plot. The phylogenetic affiliation of the module hubs and connectors is listed using the following abbreviations: *Acido*, *Acidobacteria*; *Actino*, *Actinobacteria*; *Bacter*, *Bacteroidetes*; *Chlor*, *Chloroflexi*; *Gemma*, *Gemmatimonadetes*; *Nitro*, *Nitrospirae*; *Planc*, *Planctomycetes*; *Prote*, *Proteobacteria*; Candi, Candidate. Detailed taxonomic information for module hubs and connectors is listed in Table 4.

To evaluate the possible topological roles and functions of OTUs within the W and S networks, four categories were classified based on within-module connectivity (*Zi*) and among-module connectivity (*Pi*) of each node (Fig. 6). None of the nodes were classified into the network hubs. There were 30 and 24 module hubs in the W and S networks; correspondingly, 8 and 5 connectors were observed. Both module hubs and connectors were proposed as keystone taxa due to their essential roles in network topology. The phylogenetic annotation revealed that *Proteobacteria* and *Actinobacteria*, which accounted for 65.67% of all putative keystones, would be the most prominent keystone taxa in Heshang Cave (Table 4). The prominent phylum *Proteobacteria* worked as a module hub in both of the two networks, but the genera within the phylum were distinct, such as *Beggiatoa*, *Sorangium*, and *Sphingobium* in weathered rock and *Acidovorax*, Acinetobacter, *Defluviicoccus*, *Nitrosococcus*, and *Phyllobacterium* in sediment. Interestingly, *Chloroflexi* appeared in the W network as a connector and also occurred in the S network as a module hub.

**TABLE 4:** Detailed affiliations of the keystone OTUs in the *Zi-Pi* graph

Network	Classification	No. of OTUs	Phylum	Genus
Weathered rock	Module hubs	30	*Acidobacteria* (4) *Actinobacteria* (9) *Bacteroidetes* (1) *Gemmatimonadetes* (1) *Planctomycetes* (2) *Proteobacteria* (13)	*Brevitalea* (2), unclassified (2) *Acidimicrobium* (3), *Bacterium* (3), *Microlunatus* (1), *Patulibacter* (1), *Solirubrobacter* (1) Unclassified (1) *Gemmatimonas* (1) *Planctomycete* (2) *α-Proteobacteria* (1), *Beggiatoa* (1), *Sorangium* (2), *Sphingobium* (1), unclassified (8)
	Connectors	8	*Actinobacteria* (4) *Chloroflexi* (2) *Nitrospirae* (1) Candidate (1)	*Actinobacterium* (2), *Patulibacter* (1), *Rubrobacter* (1) *Sphaerobacter* (1), unclassified (1) *Nitrospira* (1) Unclassified (1)
Sediment	Module hubs	24	*Acidobacteria* (3) *Actinobacteria* (4) *Chloroflexi* (2) *Gemmatimonadetes* (1) *Nitrospirae* (2) *Planctomycetes* (1) *Proteobacteria* (11)	*Brevitalea* (3) *Actinobacterium* (1), *Gaiella* (1), *Solirubrobacter* (1), unclassified (1) *Anaerolinea* (1), *Sphaerobacter* (1) *Gemmatimonas* (1) *Nitrospira* (1), unclassified (1) *Planctomycete* (1) *Acidovorax* (1), Acinetobacter (1), *Defluviicoccus* (1), *Nitrosococcus* (1), *Phyllobacterium* (1), unclassified (6)
	Connectors	5	*Gemmatimonadetes* (1) *Nitrospirae* (1) *Proteobacteria* (3)	*Bradyrhizobium* (1) *Nitrospira* (1) *Bradyrhizobium* (1), unclassified (2)

### Prediction of potential functions.

**FIG 7:**
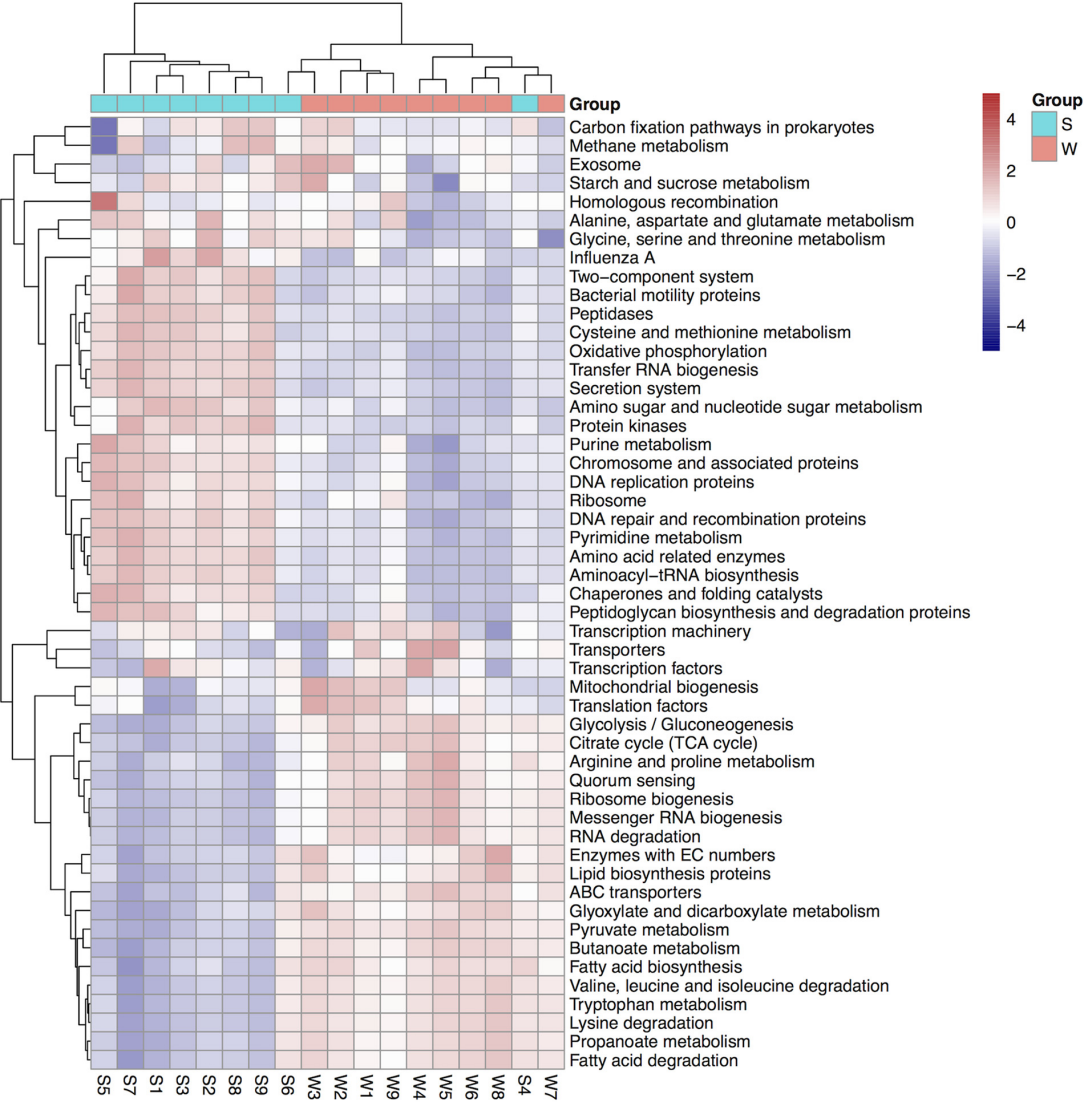
Heat map of the top 50 KEGG level-3 functional pathways for communities from weathered rock and sediment. The normalized relative abundance of each KEGG pathway is indicated by a gradient of color from purple (low abundance) to red (high abundance).

Based on statistical analysis of metagenomic profiles (STAMP), the community functions of weathered rock and sediment related to carbon/nitrogen metabolism were significantly different. For example, “carbohydrate metabolism” occupied a significantly higher proportion in weathered rock (*P* < 0.05), while “carbon fixation” and “nitrogen metabolism” showed higher proportions in sediment (Fig. S4). In detail, a heat map of the top 50 KEGG level-3 pathways for communities exhibited distinct functions in weathered rock and sediment (Fig. 7). Bacterial functions related to secretion and the signal transduction system, such as the two-component system and exosome and bacterial motility proteins, which play roles in a wide range of physiological processes, were more actively involved in sediment than in weathered rock. For carbon metabolism, the weathered rock samples maintained potential degradation functions, such as amino acid degradation and fatty acid degradation. Additionally, the sediment samples showed higher relative abundances in carbon fixation and “methane metabolism.” In addition, “glycolysis/gluconeogenesis,” “pyruvate metabolism,” “citrate cycle (TCA cycle),” and “glyoxylate and dicarboxylate metabolism” were more prevalent in weathered rock, while “oxidative phosphorylation” was abundant in sediment.

## DISCUSSION

### Microbial distinct taxonomic categories in weathered rock and sediment of Heshang Cave.

In general, the constant physicochemical conditions and limited nutrient sources over long periods in the subsurface support a stable ecosystem in karst caves as a part of autochthonous microbial endokarst communities (AMEC) (31). Compared with weathered rock, the microbial communities in sediment presented significantly higher alpha diversities. This was probably due to the relatively abundant nutrition from the outside environment by the flow of intermittent stream, which has been demonstrated in previous work (37). *Proteobacteria* thrived as the most abundant phyla in the cave, followed by *Actinobacteria* (especially the genus *Crossiella*) and *Acidobacteria* (genus *Brevitalea*) in weathered rock and sediment, respectively. The dominant populations in Heshang Cave were in accordance with previous results in a series of Kartchner caverns (12), indicating that cave-adapted microorganisms have similar bacterial lineages (10, 38). It has been shown that 61% of *Actinobacteria* isolates can produce mineral crystals in culture medium (14, 39), so the dominant *Actinobacteria* in weathered rock may directly precipitate minerals as part of their metabolic activities. *Acidobacteria* dominated the sediment, which agrees well with the theory that neutrophils preferentially colonize at highly buffering carbonate rock, while acidophiles select nonbuffering quartz (40).

Besides the habitat specificity of weathered rock and sediment, microbial spatial heterogeneity was also observed. The community composition and Shannon index of S4, S5, and S6 differed from other sediment samples, indicating that the stone flower pool shaped community diversity by changes in the flow directions of the intermittent stream. In such a relatively isolated system, microbial distribution characteristics along with the cave depth directly reflect the adaptability of species to the specific environment. In weathered rock, the dominant genera of *Rubrobacter*, *Actinobacterium*, *Brevitalea*, and *Gaiella* were replaced by groups that adapted to dark, such as *Euzebya* and *Nitrosococcus* (Fig. S3 in the supplemental material). It was believed that the *Euzebya* affiliation with *Actinobacteria* was universal in a dark cave and acts as the dominant metabolically active components from RNA clone library analyses (41). *Nitrosococcus* phylogenetically belongs to *Proteobacteria*, and their optimum growth condition in a minimal salt medium is in the dark without agitation (42), which is consistent with the cave habitat. In sediment, most of the dominant genera took on parabola curves with the cave depth. Interestingly, the lowest abundances of the predominant genera (compared with weathered rock) (Fig. 1b), such as *Brevitalea* and *Anaerolinea*, appeared in the cave middle sites. However, the less favorable *Solirubrobacter* presented the highest abundance in the middle areas. There was no denying that the flow direction of the intermittent stream affected the local accumulation of small amounts of nutrients and gave rise to the distribution shift of dominant genera in such an oligotrophic environment.

The alpha and beta diversity metrics provided a comprehensive display of community characteristics in Heshang Cave. Although microbial communities from weathered rock and sediment in the cave have spatial associations with their geological locations, the samples belonging to W and S were clustered separately, sharing similar diversity and composition, respectively. Consequently, different habitats in the karst cave were important drivers in shaping microbial communities, and this gained our interest in studying microbial interactions in weathered rock and sediment habitats.

### Abundant taxa rely on promotion effects for survival in Heshang Cave.

A previous study confirmed that cave bacterial communities may have more interactions within the niche instead of outside of it, as evidenced by construction of a comprehensive network using different sample types from eight caves (37). However, the inner microbial interactions within the certain habitat of weathered rock or sediment remained unknown. In the undeveloped Heshang Cave with little human interference, microorganisms may take different strategies to survive in the nutrient-limited ecosystem. Their interactions were determined by the fundamental drive of each species to promote their survival and played important roles in community stability, even more than species richness and abundance (21, 27). According to major topological characteristics (Table 3), the two RMT-based networks were robust in network structure and accurate for correlation threshold determination. Therefore, the constructed networks were suitable to study the interaction modes of cave microorganisms.

The sediment network connection was much higher than that of the weathered rock network connection, indicating that the interactions between species were more complicated in sediment. In addition, a large module (189 nodes) was presented in the sediment network, indicating that more microorganisms tended to inhabit the cave sediment in a modular manner. Modularity reflects the nonrandom patterns of microbial interaction and ultimately contributes to the complexity of ecological networks (43). A previous study about cave microbial networks also presented the modularized regions and even combined the different niches of the caves (37). Consequently, the sediment harbored a large and complex microbial community that remained stable over a long time.

The dominant taxa basically coexisted in networks, especially *Actinobacteria* (142 nodes, 26.49% of the total) in weathered rock and *Proteobacteria* (157 nodes, 29.13% of the total) in sediment. This agreed well with the previous result that network relationship was an important factor affecting the specific microbial abundance and community functional diversity (44, 45). Positive (cooccurrence) and negative (coexclusion) links were classified as two main interaction types (46). Mutualism and commensalism were reflected by positive links, while parasitism, antagonism, and predation were reflected by negative links (26). In total, more positive connections were detected in both of the two networks, indicating that microorganisms mainly relied on promotion effects rather than inhibition effects to sustain their survival in the oligotrophic cave. In particular, the interrelationship patterns of the abundant taxa in weathered rock and sediment were presented in the subnetworks. The species were inherently self-centered to seize the limited resources in Heshang Cave, but promotion effects enabled them to coadapt to the oligotrophic environment over the long term. The collaboration with other populations improved the resistance ability of abundant taxa to adverse circumstances intrinsically. For instance, the abundant *Crossiella* in weathered rock have been reported to harbor biosynthetic pathways of novel bioactive compounds (47). Cave microorganisms exploit different metabolic pathways, including the capacity for biomineralization and rock weathering, to survive in the cave ecosystem with low nutrient input and low productivity (3). Moreover, collaboration between autotrophic and heterotrophic microorganisms contribute to their growth and metabolism and probably enhance community functions (48). For example, experimental evidence suggested that the proximity of heterotrophs to autotrophically produced organic carbon may cause immediate sequestration of carbon in caves (49, 50). *Acidobacteria* cells were always associated with the potentially chemolithoautotrophic epsilon- or gammaproteobacterial filaments, suggesting a lifestyle based on heterotrophy or chemoorganotrophy in caves (51). The heterotrophic *Solirubrobacter*, which have been recognized as outcompeted members in the changed soil (52), probably enabled the development of other taxa in weathered rock. Although the microbial promotion effects in supporting community function was controversial, the evidence provided by cave microorganisms still advocates this view, which was in accordance with a previous study (53). In summary, the abundant taxa established positive relations with other members in the community to eliminate the detrimental effects of the oligotrophic environment.

### Keystones contributed to the ecological functions of bacterial communities.

Identifying the key species in a community is one of the most important aspects in microbial ecology, especially for communities with high diversity and complexity (23, 54). The essential role of keystones in maintaining community stability has been demonstrated based on their network topological features (55). We further explored the potential direct effects on their respective roles in ecosystem functioning. Interestingly, more module hubs and more connectors were identified in the W network than in the complicated S network (Table 4), indicating that the keystones in weathered rock made more contributions in maintaining diverse ecological functions. *Nitrosococcaceae* wb1-P19, the uncultured *Rokubacteriales*, and the uncultured *Gaiellales* member were identified as the top three keystone species in eight caves of southwest China, which might play critical roles in carbon, nitrogen, and sulfur biogeochemical cycles (37). According to the functional prediction, weathered rock was prominent in biodegradation, as reported in previous work (30). As expected, *Actinobacterium* and *Acidimicrobium* drove the communities by connecting other bacterial members, playing critical roles in organic matter decomposition and secondary metabolite production in weathered rock (41). *Acidimicrobium* have also been reported to utilize minerals such as iron and manganese (56). *Beggiatoa*, *Sorangium*, and *Sphingobium* act as gatekeepers in weathered rock, with distinct contributions to cave biogeochemical cycling. A previous study demonstrated that ammonia- and nitrite-oxidizing bacteria may be major primary producers in Movile Cave (57). *Nitrospira* have been reported as autotrophic representatives that are capable of carbon fixation and two-step nitrification (58). The chemolithoautotrophic taxa *Nitrospira* showed more cooccurrence patterns in sediment, suggesting that they played an essential role in sustaining the primary production of Heshang Cave. Additionally, more *Nitrospira* worked as keystones in sediment, which was in accordance with the CO_2_ fixation-coupled ammonia oxidation process in other cave ecosystems (16, 59). These results are consistent with predicted community functions, where prokaryote carbon metabolism showed a relatively higher abundance in sediment. In addition, more unclassified units in *Proteobacteria* also maintained complex and diverse functions in caves (60). A previous study claimed that the disappearance of key species may cause the network and modules within to break apart (61). In Heshang Cave, the role of key generalists seemed more prominent in maintaining community ecological functions because of their high abundances, diverse taxa, and complementary interactions.

In summary, the cave seclusion was inhabited by diverse microorganisms, and their spatial variations were perceptible. The microbial communities of weathered rock and sediment differed from each other significantly. The cooccurrence network revealed that both of the two network structures were nonrandom and unlikely to change substantially; in particular, the microorganisms in sediment preferred to inhabit the cave in a modular manner. Subnetwork analysis offered a particular perspective that the abundant taxa depended on more positive relations rather than negative interactions to adapt to the oligotrophic environment. In addition, the keystone species might play important metabolic roles in community ecological functions, especially degradation in weathered rock and carbon/nitrogen fixation in sediment. These results provide an improved understanding of the interactions and ecological functions of microbial communities in a cave system.

## MATERIALS AND METHODS

### Sampling and physicochemical analysis.

The undeveloped Heshang Cave is located in Qingjiang Valley, Hubei Province, central China (29°40′-30°48′N, 108°30′-111°20′E). The 250-m-long cave is rarely impacted by human activities, with an intermittent stream swerving in the middle of the cave by the stone flower pool (Fig. S1 in the supplemental material). Two main habitats, weathered rock (W) and sediment (S), were selected at nine sites along the cave depth. Samples of W and S from each sampling site were collected by a five diagonal point sampling method. The sampling depth was less than 1 cm, and each sample was homogenized. Physicochemical parameters, including pH, TOC, and concentration of dissolved cations (Ca^2+^, Mg^2+^, K^+^, Na^+^, NH_4_^+^) and anions (Cl^−^, NO_2_^−^, NO_3_^−^, SO_4_^2−^) in deionized water (1:1 [wt/vol]) were measured as described in our previous work (36).

### DNA extraction and sequencing.

Total DNA was extracted using a PowerSoil DNA kit (MoBio Laboratories, USA). The DNA quality was examined with 1.0% (wt/vol) agarose gel electrophoresis and a NanoDrop ND-1000 spectrophotometer (NanoDrop Technologies, Wilmington, USA). The bacterial V4 region of the 16S rRNA gene was amplified on Applied Biosystems (ABI) Veriti 96-well fast thermal cyclers with the primers 520F (5′-AYTGGGYDTAAAGNG-3′) and 802R (5′-TACNVGGGTATCTAATCC-3′) (62). The PCR products were purified using an EZNA gel extraction kit (Omega Bio-Tek). Reads were obtained from a MiSeq sequencing platform (Illumina, San Diego, CA, USA).

The raw sequencing data were filtered and analyzed with QIIME version 1.7.0. Clean reads were combined to generate tags by FLASH after trimming, and the tags were clustered to operational taxonomic units (OTUs) by UCLUST at a 97% similarity level (63). The representative sequences of each OTU were taxonomically classified using RDP (64).

### Statistical analysis.

Shannon-Wiener index (*H*), Pielou evenness index (*J*), and OTU richness for each sample were calculated using the vegan package in R. Comparisons of microbial diversity between the two habitats were examined by analysis of variance (ANOVA). The response ratios of treatment to control were used to compare the relative microbial abundances of the sediment to weathered rock at the phylum level and genus level. Nonmetric multidimensional scaling (NMDS) analysis was performed based on a Bray-Curtis (BC) distance matrix. Plots of linear and nonlinear regression were analyzed in R to reveal the microbial community changes along with the cave depth. A Venn plot was visualized using the VennDiagram package.

### Network construction and keystone identification.

Phylogenetic molecular ecological networks (pMEN) were constructed for weathered rock and sediment communities based on OTU relative abundances (46). Only OTUs that were detected in 6 of 9 weathered rock or sediment samples were used for network construction. Random matrix theory (RMT) was used for network construction to automatically identify the appropriate similarity threshold (*St*) and determine topological properties (21). All the steps were done at the following location: http://ieg2.ou.edu/MENA/ (molecular ecological network analysis pipeline). The basic network properties were measured based on the adjacency matrix, and networks were visualized by Cytoscape 3.5.1, similar to that in the previous study (23).

A module is when several OTUs (node in network) are highly connected within a group but less connected with outside of the group. A simulated annealing algorithm was proposed to detect the network modules. Modular structures were defined with a threshold of modularity (*M*) of >0.4. Within-module connectivity (*Zi*) and among-module connectivity (*Pi*) were used to determine the connectivity of each node, which represented their topological roles in the network. Based on *Zi* and *Pi*, the nodes were classified into four different roles: module hubs (*Zi *> 2.5 and *Pi *≤ 0.62; nodes that are highly connected within modules), network hubs (*Zi *> 2.5 and *Pi *> 0.62; nodes that are highly connected within the entire network), connectors (*Zi *≤ 2.5 and *Pi *> 0.62; nodes that connect modules), and peripherals (*Zi *≤ 2.5 and *Pi *≤ 0.62; nodes that have a few links within modules). The connectors, module hubs, and network hubs defined as generalists acted as keystone taxa in the microbial community.

### Community functional prediction.

The metabolic function profiles of the cave microbiome were predicted using the Tax4Fun package in R 3.5.1 (65). Heat maps of the KEGG level-3 functional pathways were generated in R. Statistical analysis of metagenomic profiles (STAMP) was used to analyze the differential metabolic profiles of carbon and nitrogen between the weathered rock and sediment communities (66).

### Data availability.

All sequence data were deposited in the NCBI SRA with the accession number SRP068087.
