# Supplementary material for: Microbial Interactions Drive Distinct Taxonomic and Potential Metabolic Responses to Habitats in Karst Cave Ecosystem
Source: Microbiol Spectr. 2021 Sep 8;9(2):e01152-21. doi: 10.1128/Spectrum.01152-21 (PMC8557908; doi:10.1128/Spectrum.01152-21)
Supplement: SUPPLEMENTAL FILE 1 — Supplemental material. Download Spectrum.01152-21-s0001.pdf, PDF file, 1.6 MB [file spectrum.01152-21-s0001.pdf]

Supplementary Information for

Microbial Interactions Drive Distinct Taxonomic and Potential Metabolic Responses to Habitats in Karst Cave Ecosystem

Liyuan Ma,[a] Xinping Huang,[a] Hongmei Wang,[a,b,#] Yuan Yun,[a,c] Xiaoyu Cheng,[a] Deng Liu,[a,b] Xiaolu Lu,[a] Xuan Qiu[b]

a. *School of Environmental Studies, China University of Geosciences, 430074 Wuhan, China*

b. *State Key Laboratory of Biogeology and Environmental Geology, China University of Geosciences, 430074 Wuhan, China*

c. *College of Life Sciences, Nankai University, 300071 Tianjin, China*

[*] **Corresponding author at:** School of Environmental Studies, China University of Geosciences, Wuhan 430074, China. *E-mail address*: wanghmei04@163.com (H. Wang)

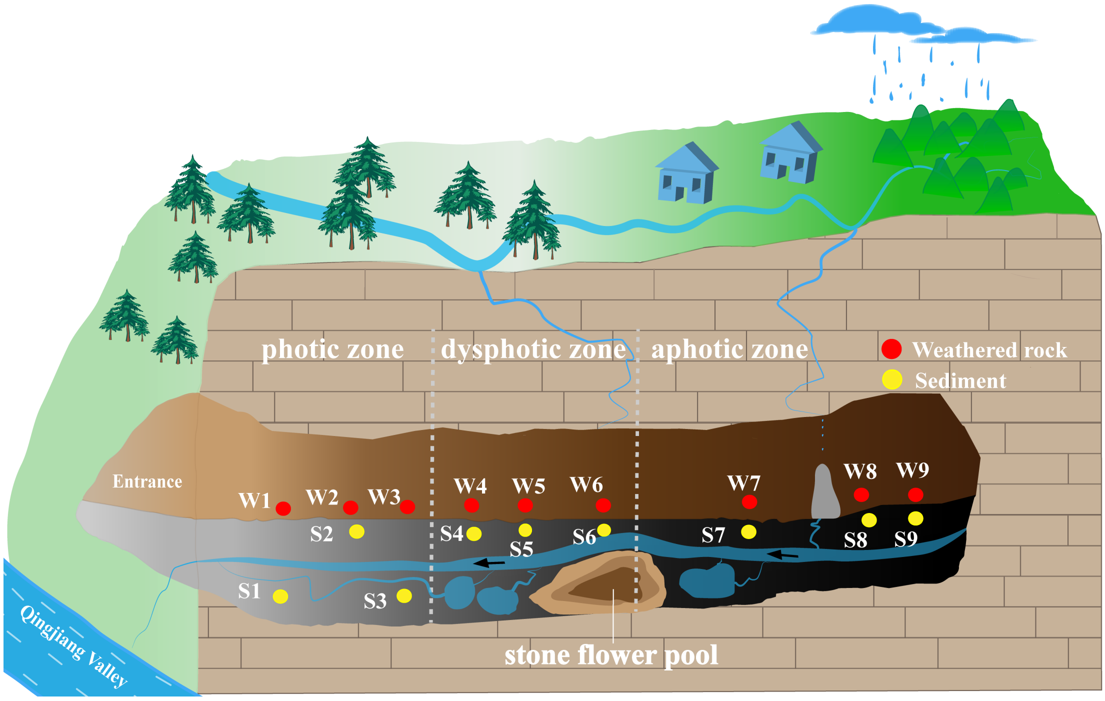

Fig. S1 Location and characters of sampling site in Heshang Cave, western Hubei Province. The cave is located above the Qingjiang Valley with little human interferences; The sampling sites of weathered rock (W) and sediment (S) were presented in cave. In the middle of the cave, there was a stone flower pool, which changed the flow directions of the underground river.

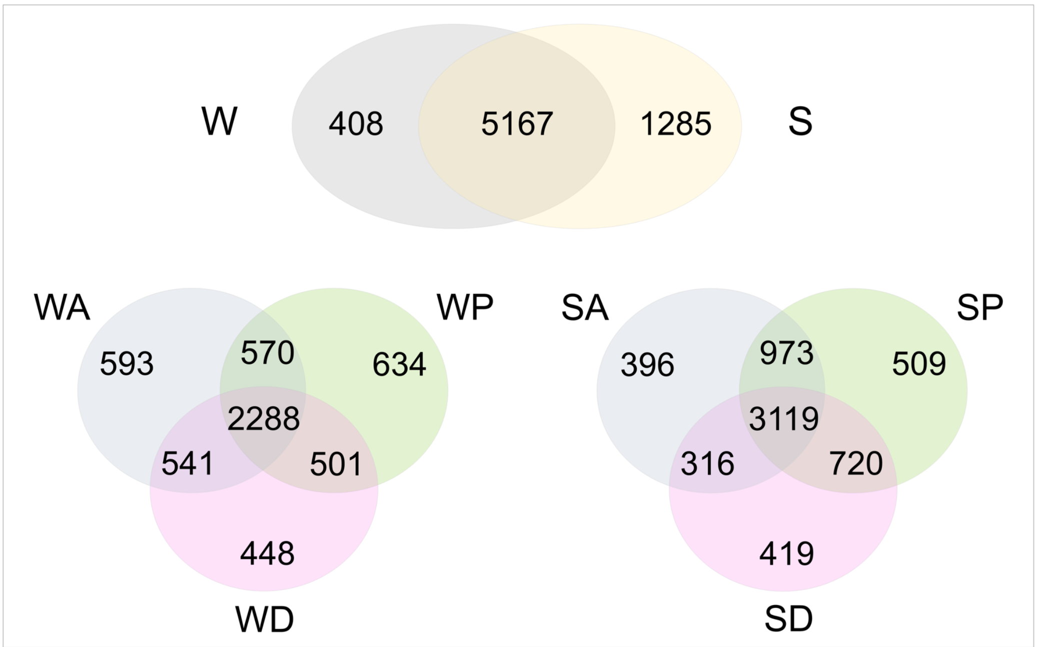

Fig. S2 VENN diagrams of OTU distribution in weathered rock (W) and sediment (S). WP, weathered rock samples from the photic entrance zone of Heshang Cave. WD, weathered rock samples from the dysphotic zone. WA, weathered rock samples from the aphotic zone. Similarly named SP, SD and SA of sediment samples.

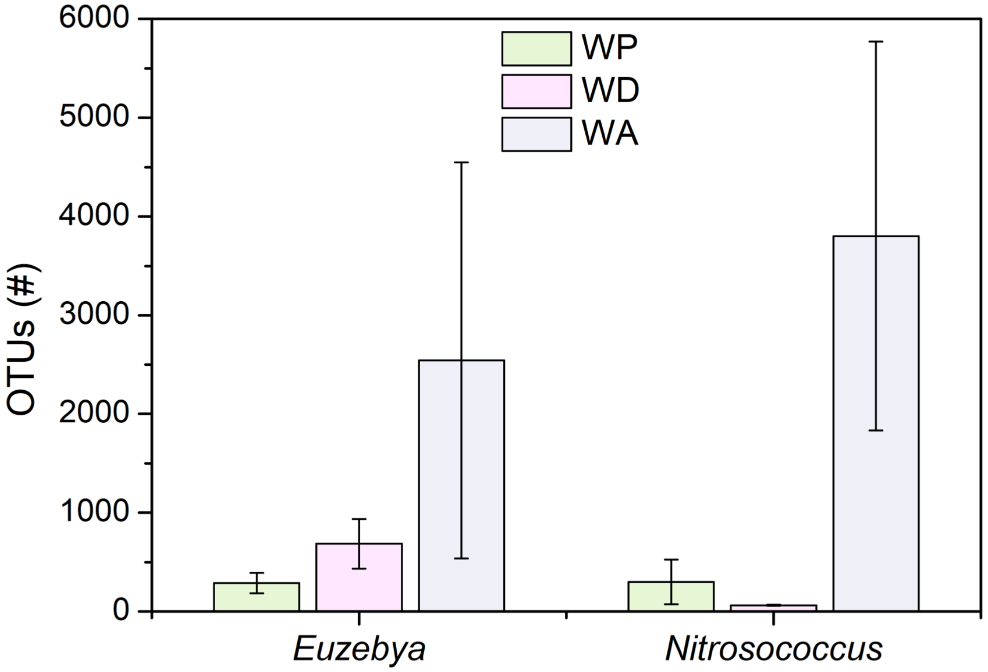

Fig. S3 The distribution of *Euzebya* and *Nitrosococcus* in WP, WD and WA in weathered rock samples. WP, weathered rock samples from the photic entrance zone of Heshang Cave. WD, weathered rock samples from the dysphotic zone. WA, weathered rock samples from the aphotic zone.

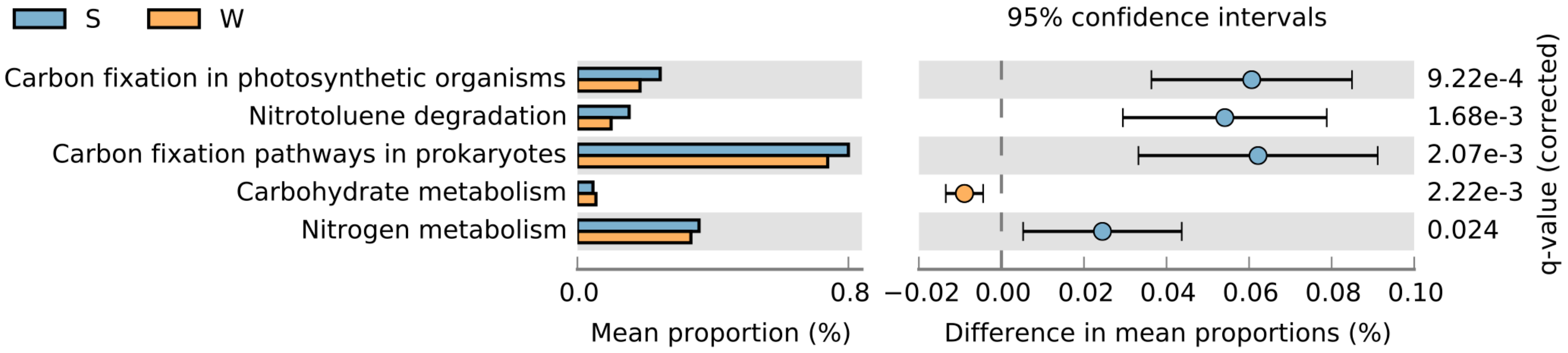

Fig. S4 The relative abundance of differential functional pathways related to carbon and nitrogen metabolism in weathered rock and sediment

Table S1 Pearson's correlation coefficient (r) between bacteria phyla and geochemical characteristics in Heshang Cave

Phyla	pH	TOC (%)	Ca^{2+}	Mg^{2+}	K^{+}	Na^{+}	NH_4^{+}	Cl^{-}	NO_2^{-}	NO_3^{-}	SO_4^{2-}
Proteobacteria	0.0557	-0.2977	-0.1514	-0.0874	**-0.3028**	-0.1589	-0.2289	-0.1941	-0.1299	-0.1005	-0.152
Actinobacteria	0.1863	0.0876	**0.4173**	**0.3203**	**0.4958**	0.2895	**0.5107**	0.2965	**0.3478**	0.2348	0.3004
Acidobacteria	**-0.4879**	**0.4599**	-0.2734	-0.1961	-0.2386	-0.2429	-0.0899	-0.2586	-0.2043	-0.2341	-0.2346
Chloroflexi	0.0892	-0.1056	0.2984	0.0897	0.0773	0.233	0.2291	0.2525	0.0642	**0.3558**	0.0153
Planctomycetes	**-0.4884**	**0.3831**	-0.0297	-0.0283	-0.1306	-0.0702	0.0513	-0.0644	-0.0442	-0.0267	-0.0846
Verrucomicrobia	**-0.7062**	**0.3964**	-0.2044	-0.1453	-0.2449	-0.254	-0.0939	-0.2347	-0.1506	-0.2374	-0.1385
Bacteroidetes	0.1705	-0.1347	-0.0479	0.0367	-0.0813	-0.134	-0.1258	-0.1859	0.0007	-0.0663	-0.0031
Nitrospirae	0.2167	-0.0296	-0.1425	-0.1035	-0.2071	-0.0027	-0.1513	0.0127	-0.1504	-0.0055	-0.1719
Gemmatimonadetes	**0.5326**	-0.1247	0.203	0.2289	0.2292	0.2692	0.2215	0.2401	0.297	**0.3232**	0.1177
Firmicutes	**0.4606**	-0.2406	**0.398**	0.2406	-0.0429	0.235	0.1824	0.2924	0.1242	**0.4346**	0.1453
Candidate	0.1489	0.0063	-0.0611	-0.0777	0.0218	-0.0918	0.0846	-0.1343	0.011	-0.0011	-0.1237
Thermotogae	0.1513	-0.1514	-0.0864	-0.0496	-0.1092	0.1266	-0.1485	0.1809	-0.0821	-0.0413	-0.088

*Significant differences (P<0.05) are indicated in bold.
